# Correlation of hepatitis C genotype with HCV-RNA, RBC-related parameters, and blood platelet count

**DOI:** 10.1097/MD.0000000000043237

**Published:** 2025-07-11

**Authors:** Jing Zhao, Yanrong Li, He Liang, Ai Feng, Lingyun Hui

**Affiliations:** a Dingxi People’s Hospital, Dingxi, Gansu Province, China; b Department of Laboratory Medicine, The First Affiliated Hospital of Xi’an Jiao tong University, Xi’an, Shaanxi Province, China; c College of Basic Medicine, Gansu University of Chinese Medicine, Lanzhou, Gansu Province, China.

**Keywords:** blood platelet count, genotype, hepatitis C, red blood cell count, virus RNA

## Abstract

**Background:**

This study aimed to clarify the correlation of hepatitis C genotype with hepatitis C virus (HCV)-RNA, red blood cell (RBC)-related parameters (i.e., RBC count, hemoglobin concentration [HGB], mean corpuscular volume, RBC distribution width-standard deviation, RBC distribution width-coefficient of variability) and blood platelet (PLT) count, providing a reference basis to clinicians for diagnosing and treating hepatitis C.

**Methods:**

Hepatitis C genotyping, RBC-related parameters, PLT count, hepatitis C virus ribonucleic acid (HCV-RNA) logarithmic values (lg) and HCV antibodies (anti-HCV; ratio of the measured value to the critical value [S/CO]) were detected in 168 patients with chronic hepatitis C.

**Results:**

Compared with the non-type I group, differences were found in HCV-RNA (lg) and anti-HCV in the type I group (*P < *.05). Compared with the negative group, significant differences were noted in anti-HCV (S/CO) in the type I and non-type I groups (*P* < .01). The RBC count and HGB of the type I group were significantly lower than those of the non-type I and negative groups. The PLT count of the 3 groups increased sequentially, with all groups exhibiting significant differences (*P* < .05). HCV-RNA (lg) was significantly negatively correlated with RBC count, HGB and PLT count (*P < *.05). The analysis of the receiver operating characteristic curve and its area under the curve indicates that RBC count, HGB, mean corpuscular volume reciprocal and PLT count exhibit certain clinical reference value for determining whether HCVs are replicating.

**Conclusion:**

Clinicians should closely monitor changes in blood cell-related indicators before and after antiviral treatment and then develop personalized treatment plans for patients.

## 1. Introduction

According to World Health Organization statistics, about 58 million people are infected with the hepatitis C virus (HCV) throughout the world; among them, 3.2 million are adolescents and children. In 2022, approximately 242 000 people worldwide died from hepatitis C, mostly due to cirrhosis and hepatocellular carcinoma (HCC).^[1]^ China has a high incidence of chronic hepatitis C (CHC), with more than 10 million patients in the country infected with this disease. The incidence rate is also increasing annually, and the infection rate is about 3.2%.^[2]^ In accordance with the data published by Polaris Observatory HCV Collaborators, an estimated 9.487 million individuals in China were infected with HCV in 2020.^[3]^ At present, no effective hepatitis C vaccine is available.

HCV is the pathogen that causes hepatitis C. It is a member of the genus *Hepatovirus* in the *Flaviviridae* family. It exhibits evident hepatotropism, and its genome is a single positive stranded RNA,^[4]^ which can cause acute hepatitis C and CHC. The lesion can cause swelling, apoptosis and necrosis of liver cells. The inflammatory zone largely involves infiltration of lymphocytes and macrophages, accompanied by the proliferation of liver cells, Kupffer cell fibroblasts and other cells. Severe liver diseases, such as cirrhosis, HCC and liver failure, can lead to death, seriously threatening human health; thus, these diseases have become prominent public health issues worldwide.

Despite extensive research on HCV’s virological and clinical manifestations, the relationship between HCV genotypes and haematological parameters remains underexplored, particularly in diverse populations. Previous studies have focused on hepatic outcomes (e.g., cirrhosis, HCC), whilst systemic effects, such as haematological abnormalities, are frequently overlooked. For example, Bagheri et al^[5]^ identified red blood cell (RBC) and blood platelet (PLT) indices as early biomarkers for HCV infection, but their association with specific genotypes was not addressed. Similarly, Lishnevska and Chemych^[6]^ reported haematological changes during antiviral therapy but did not stratify findings by genotype, leaving a critical gap in understanding genotype-specific haematological signatures.

The coexistence of viral antigens and antibodies, a phenomenon that is well-documented in studies on hepatitis B virus (HBV), further underscores the need for nuanced HCV research. Hashim et al^[7]^ demonstrated that HBV antigen–antibody coexistence (e.g., HBsAg/anti-HBs) correlates with distinct clinical and haematological profiles amongst Sudanese patients, suggesting that similar mechanisms might exist in HCV. Parallel findings from Pu et al^[8]^ and Jiang et al^[9]^ in HBV populations highlighted the clinical relevance of such immunological complexities; however, analogous investigations on HCV are scarce. Notably, HCV genotype-specific variations in immune evasion (e.g., core protein interactions with erythropoietin) can differentially alter hematopoiesis. However, this hypothesis remains untested.

Our study addresses these gaps by systematically analyzing genotype-dependent variations in HCV-RNA viral load, RBC parameters and PLT count amongst Chinese patients, a population with high genotype diversity (e.g., prevalent genotypes 1b and 2a). By integrating the findings from HBV studies (e.g., Hashim et al^[7]^) and HCV haematological research (e.g., Bagheri et al^[5]^), we provide novel insights into how genotype influences extrahepatic manifestations. This approach not only refines prognostic tools but also informs personalized monitoring strategies, particularly for high-risk genotypes (e.g., type 1’s association with severe thrombocytopenia).

Hepatitis C patients and asymptomatic carriers are the primary sources of HCV infection, with a higher degree of CHC.^[10]^ HCV can exert a serious effect on human hematopoietic system during the immune response process, leading to abnormalities in RBC-related parameters and PLT count in the fluid of patients with hepatitis C and latent infections.^[11]^ The early diagnosis of hepatitis C requires a combination of clinical symptom detection for HCV antibodies (anti-HCV) and HCV-RNA in serum, and hepatitis C genotype.^[12]^ Further detection of HCV-RNA after positive screening for anti-HCV in patients is recommended as the preferred diagnostic approach for HCV infection.^[13]^ HCV-RNA is a quantitative detection method for HCV. It is a diagnostic tool for current HCV infection, and it can monitor the effectiveness of antiviral treatment. HCV-RNA exhibits the characteristics of accuracy and speed.^[10]^ Various genotypes of hepatitis C have different clinical indicators, symptoms and treatment plans. The severity of type I hepatitis C genotype is considerably greater than those of other genotypes.^[14]^ Patients with CHC can understand the progression and severity of their condition by combining routine blood indicators.^[15]^

The current study analyzed the relationship between different hepatitis C genotypes and indicators, such as HCV-RNA, RBC-related parameters and PLT count, in the blood of 168 patients with hepatitis C admitted to the Department of Infectious Diseases at the First Affiliated Hospital of Xi’an Jiaotong University, providing a reference for the treatment of early hepatitis C.

## 2. Materials and methods

### 2.1. Research participants

This study performed an analysis of 168 untreated CHC patients in the Department of Infectious Diseases, First Affiliated Hospital of Xi’an Jiaotong University from January 2018 to May 2021, including 78 males and 90 females, aged 21 to 77 years, with an average age of (54.72 ± 13.23) years. All cases were selected in accordance with the diagnostic criteria for CHC in the Guidelines for the Prevention and Treatment of Hepatitis C (2022 Edition) established by the Hepatology Branch and Infectious Diseases Branch of the Chinese Medical Association,^[16]^ with positive anti-HCV and HCV-RNA ≥ 5.0 × 10^1^ IU/mL. The excluded cases were as follows: Pregnant or lactating women; complicated with hepatitis A, hepatitis B, hepatitis D, hepatitis E and other viral infections; combined with human immunodeficiency virus, Epstein–Barr virus, human cytomegalovirus and other viral infections; chronic liver disease caused by other factors, including immune hepatitis, drug-induced hepatitis, cirrhosis, alcoholic liver, fatty liver, liver transplant, HCC and liver damage; and patients with reduced blood system diseases and incomplete cases in the tertiary system.

Patients were consecutively enrolled during the study period to minimize selection bias. All HCV-RNA and genotyping tests were performed at the Central Laboratory of the First Affiliated Hospital of Xi’an Jiaotong University, which participates in quality control programmes to ensure assay reliability. All the patients in this study were informed and had signed informed consent forms, which were reviewed and approved by the hospital’s medical ethics committee.

### 2.2. Instruments and reagents

The SYSMEX XN9000 blood cell analyzer, supporting reagents and quality control products were acquired from SYSMEX Company (Japan). The ABI7500 fluorescent polymerase chain reaction (PCR) amplifier was obtained from ABI Corporation. The hepatitis C genotyping test kit and HCV nucleic acid quantitative test kit were from Da’an Gene Co., Ltd. and Sun Yat-sen University (China). Abbott i2000SR chemiluminescence assembly line and supporting reagents (standards) were from Abbott Laboratories.

### 2.3. Experimental methods

In the morning whilst at rest, the patients were subjected to standardized blood collection, and 2 mL of blood was extracted using EDTA-K_2_ anticoagulant tubes. The collected blood was mixed upside down to ensure that the specimen would be free of clots. After mixing, complete blood cell count was performed to detect RBC-related parameters and PLTs. Then, 3 mL of blood was collected in 2 vacuum-drying tubes and centrifuged at 4000 r/min for 10 minutes with a low-speed centrifuge to separate the serum. Subsequently, anti-HCV, HCV-RNA and hepatitis C genotype were detected. The PCR fluorescence probe method was used to detect HCV-RNA and perform hepatitis C genotyping. HCV-RNA extraction was based on the magnetic bead method. Then, reverse transcription quantitative real-time PCR was performed using ABI7500 (hot cycling conditions: 50℃ for 15 minutes, 95℃ for 15 minutes; 45 cycles at 94℃ for 15 seconds and 55℃ for 45 seconds). The detection limit of HCV-RNA was 5.0 × 101 IU/mL, and HCV-RNA was analyzed using logarithmic values (lg). All the selected patients with hepatitis C were genotyped and divided into 3 groups based on the test results: type I, non-type I, and negative. Chemiluminescence particle immunoassay was used to detect anti-HCV, and the ratio of the measured value to the critical value (S/CO) was calculated, with S/CO ≥ 1 indicating positivity. The above experiments were strictly operated in accordance with the specifications provided in the reagent manual.

In accordance with the previous literature^[17]^ and on the basis of the quantitative results of HCV-RNA, HCV-RNA was divided into the low-load (5.0 × 10^1^–5.0 × 10^5^ IU/mL) and high-load (5.0 × 10^5^–1.0 × 10^8^ IU/mL) groups. HCV-RNA concentration was <5.0 × 10^1^ IU/mL. The test result showed that it was below the detection limit and belonged to the negative HCV-RNA group. Simultaneously, it met the requirement that anti-HCV (S/CO) should be positive ≥1.

### 2.4. Statistical processing

Statistical analysis was conducted using SPSS 22.0 statistical software (SPSS Inc., Chicago), and count data were presented as mean ± standard deviation ($\bar{x}\pm s$) in accordance with a normal distribution. One-way analysis of variance was used to analyze the statistical differences, and Pearson analysis was used for bivariate correlation analysis. The diagnostic ability of the detection items for diseases was compared using the receiver operating characteristic (ROC) curve and area under curve (AUC). A *P* value of <.05 was considered statistically significant.

## 3. Results

### 3.1. Comparison between HCV-RNA and anti-HCV detection results amongst patients with different hepatitis C genotypes

**Figure 1.**
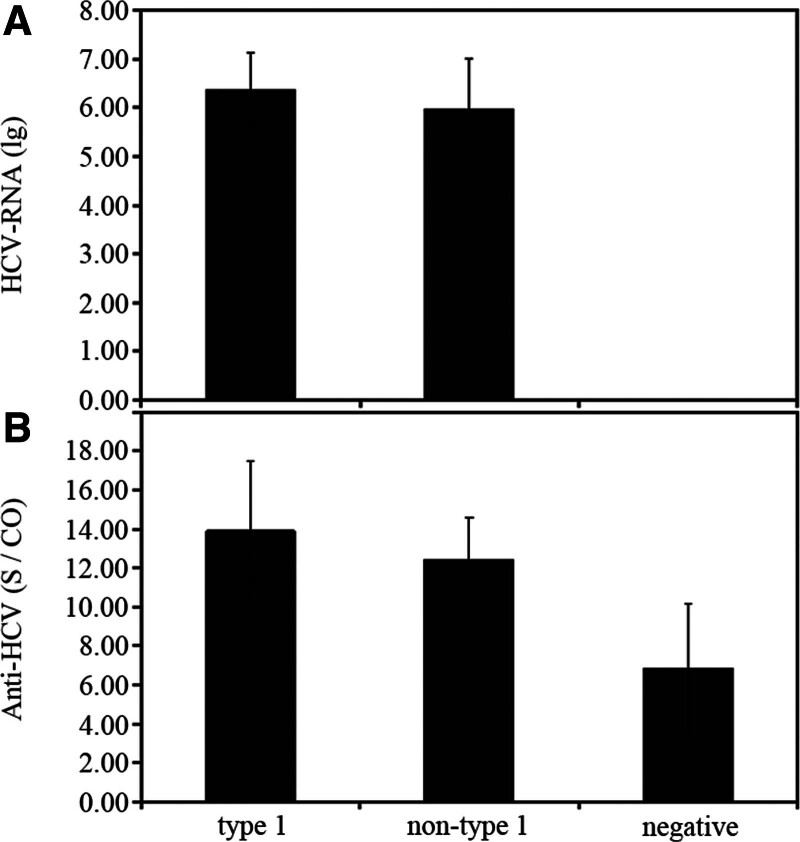
Comparison of HCV-RNA (lg) (A) and anti-HCV (S/CO) (B) levels across hepatitis C genotypes. Data were analyzed by analysis of variance. Anti-HCV = hepatitis C virus antibodies, HCV-RNA = hepatitis C virus ribonucleic acid.

The HCV-RNA (lg) value in the type I group was higher than that in the non-type I group, and the HCV-RNA (lg) value in the negative group was lower than the detection limit. There was a difference in HCV-RNA (lg) between the type I group and the non-type I group (Fig. 1A, *P = *.0434). The anti-HCV (S/CO) values of type I group, non-type I group and negative group decreased sequentially; there was a difference in anti-HCV (S/CO) between the type I group and the non-type I group (Fig. 1B, *P = *.0429); compared with the negative group, there were significant differences in anti-HCV (S/CO) in the type I group and the non-type I group (Fig. 1B, *P = *.000, <.01).

### 3.2. RBC, HGB, MCV, RDW-SD, RDW-CV and PLT count analysis of patients with different hepatitis C genotypes

The RBC count and hemoglobin concentration (HGB) of type I group were significantly lower than these of non-type I and negative groups, compared with those of non-type I and negative groups, RBC count and HGB were statistical significance. There was no statistical significance in RBC count and HGB between non-type I and negative. The levels of mean corpuscular volume (MCV), RBC distribution width (RDW)-SD and RDW-coefficient of variability (RDW-CV) were comparable amongst type I, non-type I and negative groups, with no statistical significant. The PLT count of type I, non-type I and negative groups increased sequentially, there were statistical significance (Table 1).These findings suggest that HCV type I infection is associated with more pronounced hematological abnormalities, including reduced RBC count, lower HGB and PLT count, compared to non-type I infections and negative individuals.

**Table 1:** Comparison of laboratory results of RBC-related parameters and PLT in patients with different hepatitis C genotypes.

Genotype	RBC(×10^12^/L)	HGB(g/L)	MCV(fL)	RDW-SD(fL)	RDW-CV(%)	PLT(×10^9^/L)
Type I (n = 71)	3.90 ± 0.80	120.60 ± 25.16	92.50 ± 10.34	45.97 ± 5.60	13.61 ± 1.82	142.33 ± 85.32
Non-type I (n = 51)	4.28 ± 0.71	131.58 ± 23.14	93.10 ± 6.39	45.84 ± 6.15	13.62 ± 2.01	162.85 ± 85.85
Negative (n = 46)	4.37 ± 0.67	134.87 ± 22.09	90.30 ± 5.66	43.06 ± 3.95	13.22 ± 1.34	180.30 ± 80.09
Type I vs negative	*P = *.0116	*P = *.0467	*P = *.3968	*P = *.0626	*P = *.3962	*P = *.0336
Type I vs non-type I	*P = *.0438	*P = *.0486	*P = *.9316	*P = *.9998	*P = *.9994	*P = *.0432
Non-type I vs negative	*P = *.2641	*P = *.8479	*P = *.0558	*P = *.1160	*P = *.4306	*P = *.0193

ANOVA was used to analyze statistical differences. *P* < .05 was considered statistically significant.

ANOVA = analysis of variance, HGB = hemoglobin concentration, MCV = mean corpuscular volume, PLT count = platelet count, RBC count = red blood cell count, RDW-CV =  red blood cell distribution width-coefficient of variability, RDW-SD = red blood cell distribution width-standard deviation.

### 3.3. Analysis of the abnormal rates of RBC-related parameters and PLT count in patients with three different hepatitis C genotypes

The abnormal rate of RBC-related parameters and the PLT count in the three groups (type I, non-type I and negative) decreased successively. Analysis showed that the abnormal rates of RBC count, HGB and PLT count in the type I group were statistically significant compared with those in the non-type I group and the negative group. The RBC count, HGB and PLT count in the non-type I group were statistically significant compared with those in the negative group. The abnormal rates of MCV and RDW-SD in the type I group and the negative group were statistically significant, while compared with the type I group and the negative group, the abnormal rates of MCV and RDW-SD in the non-type I group were not statistically significant The abnormal rate of RDW-CV in the 3 groups was not statistically significant (Table 2).

**Table 2:** Comparison of abnormal rates of RBC-related parameters and PLT in patients with different genotypes of hepatitis C.

Genotype	RBC(%)	HGB(%)	MCV(%)	RDW-SD(%)	RDW-CV(%)	PLT(%)
Type I (n = 71)	72.55	56.86	3.92	5.89	9.80	45.10
Non-type I (n = 51)	45.07	38.62	1.41	2.82	9.49	33.80
Negative (n = 46)	34.78	30.43	1.35	2.17	8.97	23.91
Type I vs negative	*P < *.0001	*P = *.0087	*P = *.0415	*P = *.0356	*P = *.8421	*P = *.0201
Type I vs non-type I	*P = *.0022	*P = *.0329	*P = *.5609	*P = *.3829	*P = *1.0000	*P = *.0194
Non-type I vs negative	*P = *.0387	*P = *.0365	*P *= 1.0000	*P *= 1.0000	*P = *.8253	*P = *.0446

ANOVA was used to analyze statistical differences. *P* < .05 was considered statistically significant.

ANOVA = analysis of variance, HGB = hemoglobin concentration, MCV = mean corpuscular volume, PLT count = platelet count, RBC count = red blood cell count, RDW-CV = red blood cell distribution width-coefficient of variability, RDW-SD = red blood cell distribution width-standard deviation.

### 3.4. Analysis of HCV-RNA with RBC-related parameters and PLT count

The levels of MCV, RDW-SD, and RDW-CV in the high-load HCV-RNA group, the low-load HCV-RNA group and the HCV-RNA negative group were comparable, not statistically significant (Table 3). The RBC count, HGB and PLT count in the high-load HCV-RNA group, the low-load HCV-RNA group and the HCV-RNA negative group increased successively. Compared with the negative group, the high-load HCV-RNA group showed statistical significance in terms of RBC count, HGB and PLT count. There was no statistical significance in RBC count, HGB and PLT count between the low-load HCV-RNA group and the high-load HCV-RNA group, as well as the HCV-RNA negative group (Table 3).

**Table 3:** Comparison of HCV load with RBC-related parameters and PLT laboratory results.

	RBC(×10^12^/L)	HGB(g/L)	MCV(fL)	RDW-SD(fL)	RDW-CV(%)	PLT(×10^9^/L)
High-load HCV-RNA group (n = 79)	3.25 ± 0.74	120.16 ± 23.38	92.21 ± 9.02	45.85 ± 6.50	13.96 ± 2.15	127.81 ± 82.38
Low-load HCV-RNA group (n = 36)	4.36 ± 0.79	131.30 ± 27.56	92.71 ± 6.12	46.92 ± 6.69	13.65 ± 2.54	170.42 ± 88.07
HCV-RNA negative group (n = 53)	4.87 ± 0.66	131.85 ± 21.85	90.31 ± 5.60	45.07 ± 3.90	13.22 ± 1.33	180.40 ± 79.22
HCV-RNA high-load vs negative groups	*P = *.0001	*P = *.0350	*P = *.1731	*P = *.1927	*P = *.2485	*P = *.0421
High vs low HCV-RNA load groups	*P = *.9936	*P = *.9699	*P = *.9890	*P = *.5868	*P = *.4723	*P = *.7833
Low-load HCV-RNA vs HCV-RNA negative groups	*P = *.4206	*P = *.8579	*P = *.2614	*P = *.5702	*P = *.1102	*P = *.0936

ANOVA was used to analyze statistical differences. *P* < .05 was considered statistically significant.

ANOVA = analysis of variance, HGB = hemoglobin concentration, MCV = mean corpuscular volume, PLT count = platelet count, RBC count = red blood cell count, RDW-CV = red blood cell distribution width-coefficient of variability, RDW-SD = red blood cell distribution width-standard deviation.

### 3.5. Correlation analysis of HCV-RNA (lg) with RBC-related parameters and PLT count

**Figure 2.**
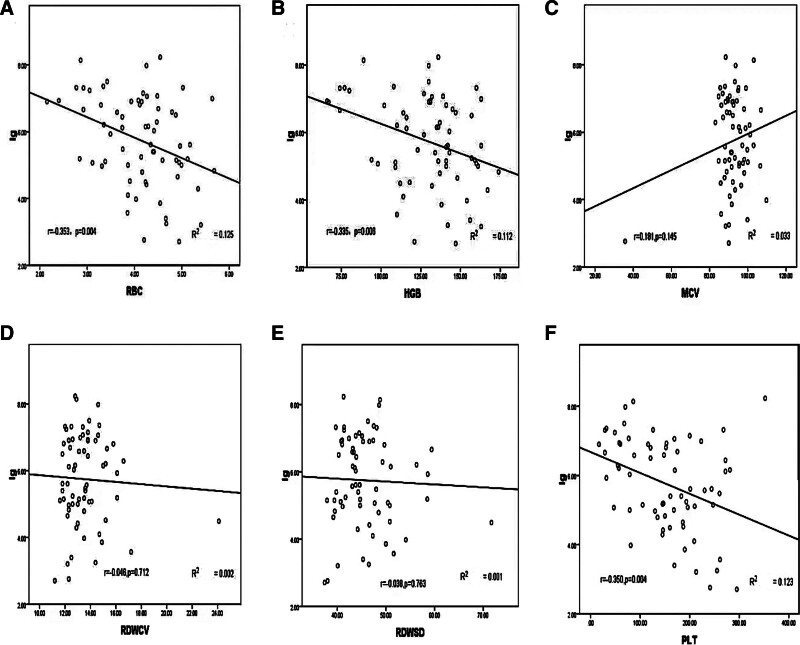
HCV-RNA(lg)and RBC-related parameters, PLT correlation linear analysis. (A) RBC count; (B) hemoglobin (HGB); (C) mean corpuscular volume (MCV); (D) RDW-CV; (E) RDW-SD; (F) platelet (PLT) count. HCV-RNA = hepatitis C virus ribonucleic acid, PLT = blood platelet, RBC = red blood cell, RDW-CV = red blood cell distribution width-coefficient of variability, RDW-SD = red blood cell distribution width-standard deviation.

In a sample of 115 HCV-RNA positive patients, the linear relationship between HCV-RNA (lg) and RBC-related parameters was compared, and HCV-RNA (lg) was determined to be significantly negatively correlated with RBC count (Fig. 2A, *r* = −0.353, *P = *.004), HGB (Fig. 2B, *r* = −0.335, *P = *.006) and PLT count (Fig. 2F, *r* = −0.350, *P* = .003). HCV-RNA exhibited a negative correlation trend with RDW-CV (Fig. 2D, *r* = −0.0046, *P* = .712) and RDW-SD (Fig. 2E, *r* = −0.0038, *P* = .763), and a positive correlation trend with MCV (Fig. 2C, *R* = 0.181, *P = *.145). However, no significant correlation was found.

### 3.6. Predictive values of RBC-related parameters and PLT count and whether HCV is replicating

**Figure 3.**
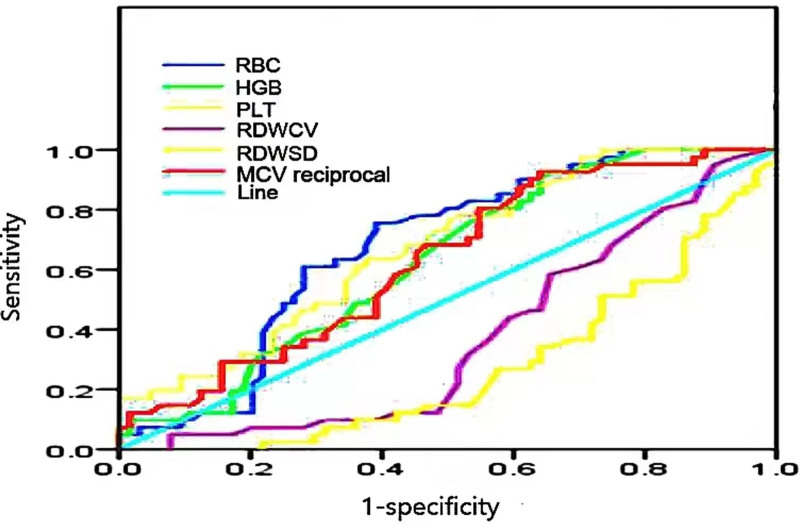
Analysis of subject operating characteristics (ROC) curve of RBC-related parameters, PLT and hepatitis C virus reproduction. RBC = red blood cell.

As indicated in Table 3, HCV-RNA (lg) is significantly negatively correlated with RBC, HGB, and PLT count. A negative correlation trend was found with RDW-CV and RDW-SD, whilst a positive correlation trend was noted with MCV. However, no significant correlation was recorded. With regard to the ROC curve, the reciprocal of MCV was used to analyze its relationship with whether HCVs are replicating. In accordance with the ROC curve analysis, the AUC of RBC count was 0.678 (*P* = .002) and that of HGB was 0.628 (*P = *.027). The AUC of MCV reciprocal was 0.630 (*P* = .025). The AUC of RDW-CV was 0.370 (*P = *.026). The AUC of RDW-SD was 0.273 (*P = *.001). The AUC of PLT count was 0.674 (*P = *.003; Fig. 3). RBC, HGB, MCV reciprocal and PLT count achieve certain accuracy in determining HCV replication, whilst RDW-SD and RDW-CV exhibit no diagnostic value (Fig. 3).

## 4. Discussion

The destruction of liver tissues by HCV exacerbates the condition and slows down the progression of the disease. In accordance with the nucleotide sequence differences of hepatitis C genes, 6 genotypes (I–VI) and over 70 subtypes of global HCV isolates exist.^[18,19]^ The HCV genotypes in China are mostly types Ib and IIa, with over 60% being type Ib. The vast majority of cities in southern China have type Ib, with type IIa exhibiting an increasing trend from south to north.^[20]^ The genotypes of hepatitis C studied in this work are type I and non-type I, and more people have been diagnosed with type I than non-type I; this finding is consistent with relevant literature reports.^[21,22]^

HCV infection exhibits a high degree of insidiousness and chronicity, and thus, it is difficult to detect and can easily transform into chronic hepatitis. The severity of hepatitis C caused by different HCV genotypes varies, and the sustained viral response rate (SVR) to drugs also differs. Therefore, HCV genotyping detection has important clinical application value, and it can serve as a guide in formulating personalized treatment plans for hepatitis C patients based on the degree of liver damage caused by HCV infection. The SVR of type I is <50%, whilst that of non-type I is 70% to 80%. The cure rate of type I is lower than that of non-type I.^[23]^ The genotype of HCV is also closely related to the level of HCV-RNA. HCV-RNA is an indicator of whether HCV is replicating, and it can be used to determine the degree of liver damage in patients and guide clinical doctors in antiviral treatment. The reduction in PLT and RBC counts is an important feature of chronic hepatitis and cirrhosis.^[24]^ PLTs represent one of the components of blood. They are small cytoplasmic fragments that have broken down from mature bone marrow megakaryocytes and entered the bloodstream through bone marrow hematopoietic tissues. They play an important role in blood coagulation and haemostasis.^[25,26]^ RBCs are also important components of blood. They are generated in the bone marrow and mostly composed of hemoglobin. RBCs can transport oxygen to the body through hemoglobin and exhibit a defensive effect against infections.^[27]^ Many factors can lead to a decrease in RBC count, HGB and PLT count in CHC caused by type I and high-load HCV-RNA. The major reasons may be as follows: Type I HCV has a high-viral-load; it is difficult to remove from the body and has a long survival time.^[28]^ Dai et al suggested that HCV may be directly related to a decrease in PLT count.^[29]^ The bone marrow is the primary site for synthesizing RBCs, and megakaryocytes in bone marrow hematopoietic tissues are the primary site for thrombopoietin (TPO).^[30]^ HCV exhibits a hepatophilic nature and can aggravate damage to liver tissues, leading to liver function impairment. HCV can also invade bone marrow stem cells, directly destroying bone marrow stromal cells and megakaryocytes, producing abnormal cytokines in the body and leading to a decrease in the differentiation, development and maturity of bone marrow megakaryocytes. Consequently, bone marrow hematopoietic function is inhibited. RBCs and TPO also decrease, causing a reduction in RBCs and hemoglobin and a decrease in PLT synthesis.^[31]^ Simultaneously, the body needs to consume a large amount of PLTs to clear HCV. Therefore, bone marrow puncture and biopsy in patients with hepatitis C can help diagnose and prevent haematological diseases related to hepatitis C. Patients with CHC have decreased coagulation function, resulting in bleeding and the non-coagulation of blood; these conditions can be considerably reduced by consuming PLTs.^[32]^ HCV infection can cause abnormalities in the autoimmune system, leading to the production of auto-PLT antibodies, abnormal hematopoietic function and cold globulinemia. Autoimmune diseases, such as autoimmune thrombocytopenia, can cause a decrease in blood cells.^[33]^ Kajiharaeta discovered GPIIb–IIIa antibodies in patients with liver cirrhosis and determined that these autoantibodies might be partially associated with liver cirrhosis thrombosis; liver cirrhosis patients associated with HCV are more likely to produce B cells that resist GPIIb–IIIa antibodies.^[34]^ The clearing out of HCV can reduce the production of autoantibodies and prolong the life span of PLTs. CHC patients with concurrent splenic hyperfunction and hepatosplenomegaly can cause monocyte proliferation, phagocytosis of PLTs and destruction of blood cells, resulting in thrombocytopenia and even anemia.^[35,36]^ However, this viewpoint is highly controversial because some patients in clinical practice still experience a decrease in PLT count after splenectomy, indicating that other factors may be contributing to the decrease in PLT count. Folic acid, vitamin B12, iron and other substances are synthesized by RBCs.^[37]^ As the disease worsens and liver cells are damaged, these hematopoietic raw materials decrease, and hemoglobin synthesis also decreases. When infected with HCV, the liver’s detoxification function is impaired, the ability to clear the virus in the body decreases and the hematopoietic environment is disrupted and worsened, leading to a poor living environment for blood cells and a decrease in blood cells.^[38]^ TPO is synthesized in liver cells and participates in the generation of megakaryocytes and PLTs by binding to TPO receptors in bone marrow megakaryocytes.^[39]^ Olson et al demonstrated that TPO mimetics can increase PLT count in patients with thrombocytopenic chronic lung disease.^[40]^ After HCV infection, the synthesis of PLTs in peripheral blood decreases as the synthesis of TPO decreases. In addition, patients with CHC exhibit abnormal PLT distribution, affecting the quantity and density of PLTs.^[41]^ Therefore, the RBC count, HGB and PLT count of HCV-RNA in the type I and high-load groups were lower than those in the non-type I and high-load groups.

The level of HCV-RNA (lg), the ratio of anti-HCV (S/CO; Fig. 1) and the abnormal values of RBC count, HGB, RDW-CV, RDW-SD, MCV and PLT count in the type I group in this study were higher than those in the non-type I group (Table 2). RBC count, HGB and PLT count in the type I group were lower than those in non-type I group, with statistical significance (*P* < .05; Table 1), indicating that the degree of liver injury in type I patients is more severe. This conclusion is similar to that in the previous literature. RBC count, HGB and PLT count in the high-viral-load HCV-RNA group were lower than those in the low-viral-load and negative groups (Table 3). HCV load was significantly negatively correlated with RBC count, HGB and PLT count (*P* < .05; Fig. 2A, B, F), indicating that HCV causes certain damage to the internal and external tissues of the liver. Type I genotype and high-viral-load HCV-RNA cause greater damage to the liver and exert an important effect on the generation and metabolism of RBCs, HGB, and PLTs, leading to their reduction. By analyzing the ROC curve, RBC count, HGB, PLT count and the reciprocal of MCV were found to have an AUC >0.5 (*P* < .05; Fig. 3) when determining the negative genotype of hepatitis C, also demonstrating certain clinical significance and diagnostic value.

Several limitations of this study should be acknowledged. Firstly, the moderate sample size (n = 168) and single-center design may limit the generalisability of our findings, particularly for underrepresented HCV genotypes (e.g., types 3–6) or populations with other comorbid conditions.^[42]^ Secondly, the cross-sectional nature of our analysis precludes causal inferences about the temporal relationships amongst HCV genotype, viral load and hematologic abnormalities. Longitudinal studies that track hematologic recovery post-direct-acting antiviral therapy are necessary to clarify these dynamics.^[43]^ Thirdly, although we controlled for major confounders (e.g., cirrhosis), unmeasured variables, such as nutritional status (iron/vitamin B12 deficiency) and concurrent medications (e.g., ribavirin), could influence cytopenias.^[37]^ Future multicentre studies with broader demographic representation and standardized protocols (e.g., bone marrow biopsies for thrombocytopenia etiology) can refine genotype-specific risk stratification.^[5]^ Despite these constraints, our findings are aligned with global data on genotype 1’s aggressive profile^[20,23]^ and underscore the clinical utility of routine hematologic monitoring in HCV management.

Despite significant progress in understanding genotype-specific hematologic abnormalities in HCV infection, several challenges remain. Firstly, the precise mechanisms that underlie genotype 1-associated cytopenia (e.g., thrombocytopenia via bone marrow suppression vs immune-mediated destruction) require further elucidation, particularly given the conflicting evidence with regard to splenectomy outcomes.^[42]^ Second, although RBC count, HGB, and PLT count demonstrate diagnostic potential for viral replication (AUC > 0.6), their utility is limited due to overlap with cirrhosis-related hematologic changes, necessitating larger multicentre studies to validate genotype-specific cutoff values. Future research should focus on the following: longitudinal monitoring of hematologic recovery post-DAA therapy to determine if genotype 1’s slower SVR is correlated with delayed PLT normalization^[43]^; integrated omics approaches that combine transcriptomics (e.g., TPO receptor expression) and proteomics to unravel genotype-driven hematopoietic dysfunction^[44]^; and point-of-care technologies that leverage machine learning to predict genotype-specific complications by using routine blood parameters, particularly in resource-limited settings.^[5]^ In addition, emerging evidence suggests that HCV may induce clonal hematopoiesis, increasing myeloid malignancy risk; this area is critical for investigation.^[45]^ Addressing these gaps will optimize personalized management and advance World Health Organization’s HCV elimination goals.

## 5. Conclusion

In summary, different genotypes of hepatitis C are closely related to HCV-RNA, RBC-related parameters and PLT count, and they can complement one another. Combined testing can help clinical doctors in better guiding patients when using antiviral drugs, develop the best treatment plan and estimate prognosis efficiently. The subtypes of HCV genes were not further classified due to limited sample size and experimental methodology, and thus, further in-depth and detailed research is necessary.

## Acknowledgments

We would like to thank Dr Benliang Deng for his advice on this work. We also like to thank the native English-speaking experts from KGSupport Company for editing our manuscript.

## Author contributions

**Conceptualization:** Jing Zhao.

**Data curation:** He Liang, Ai Feng.

**Formal analysis:** He Liang, Ai Feng.

**Funding acquisition:** Yanrong Li, Lingyun Hui.

**Writing – original draft & editing:** Jing Zhao.

**Writing – review & editing:** Yanrong Li, Lingyun Hui.
